# Multivalent Batteries—Prospects for High Energy Density: Ca Batteries

**DOI:** 10.3389/fchem.2019.00079

**Published:** 2019-02-20

**Authors:** Damien Monti, Alexandre Ponrouch, Rafael B. Araujo, Fanny Barde, Patrik Johansson, M. Rosa Palacín

**Affiliations:** ^1^Institut de Ciència de Materials de Barcelona, ICMAB-CSIC, Bellaterra, Spain; ^2^Department of Physics, Chalmers University of Technology, Göteborg, Sweden; ^3^Battery & Fuel Cell, Advanced Material Research and Development Department, Technical Centre, Toyota Motor Europe, Zaventem, Belgium; ^4^ALISTORE-ERI European Research Institute, CNRS FR 3104, Hub de l'Energie, Rue Baudelocque, Amiens, France

**Keywords:** calcium batteries, energy-cost model, Ca-sulfur, Ca-ion, metal anodes

## Abstract

Batteries based on Ca hold the promise to leapfrog ahead regarding increases in energy densities and are especially attractive as Ca is the 5th most abundant element in the Earth's crust. The viability of Ca metal anodes has recently been shown by approaches that either use wide potential window electrolytes at moderately elevated temperatures or THF-based electrolytes at room temperature. This paper provides realistic estimates of the practical energy densities for Ca-based rechargeable batteries at the cell level, calculated using open source models for several concepts. The results from the Ca metal anode batteries indicate that doubled or even tripled energy density as compared to the state-of-the-art Li-ion batteries is viable if a practical proof-of-concept can be achieved.

## Introduction

**Figure 1:**
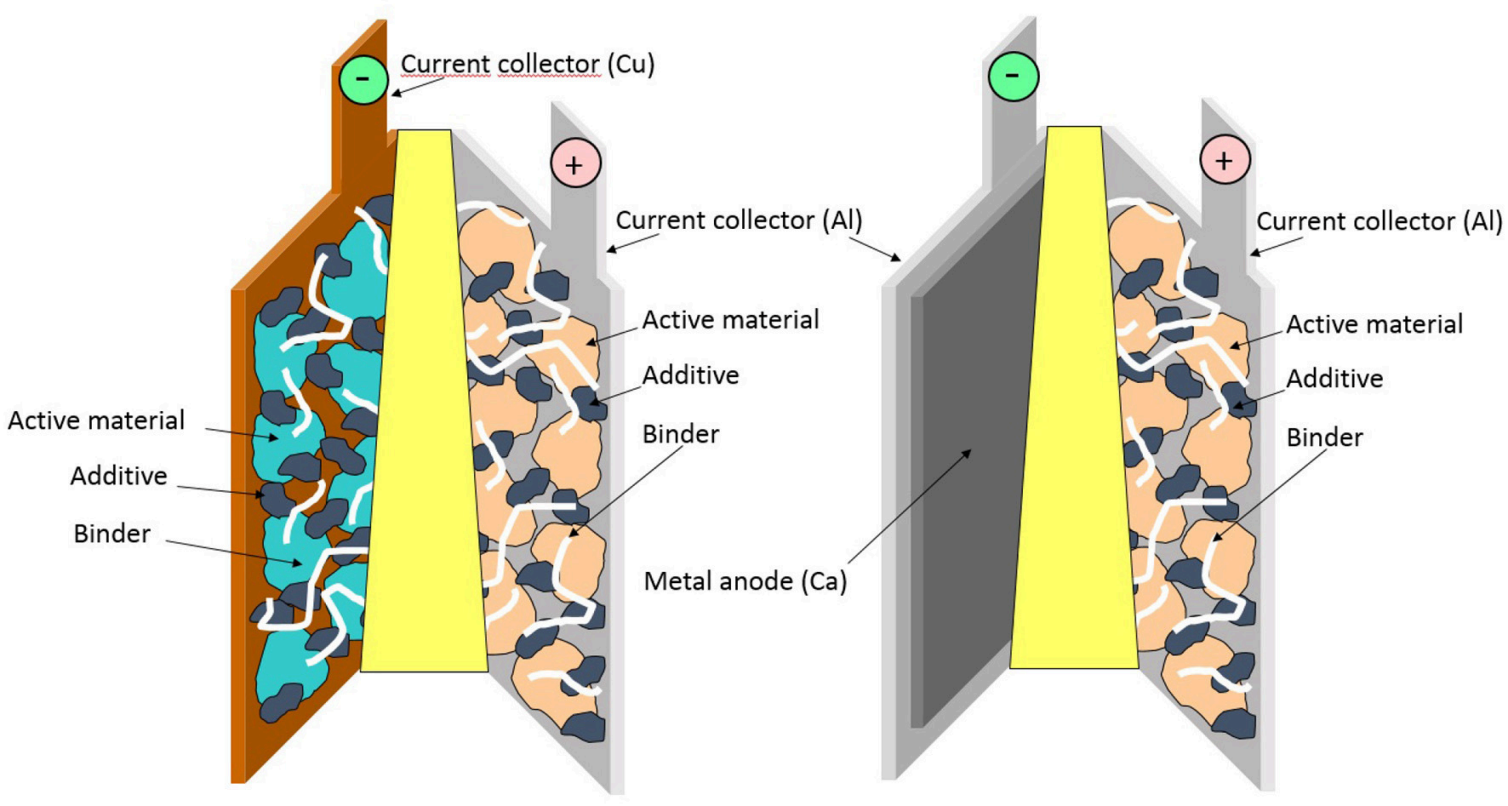
Schematic of a LIB (left) with Cu and Al current collectors and a CaB (right) with two Al current collectors. They are completed with a separator, an electrolyte, and electrodes. Each composite electrode is here composed of active material, carbon black additive, and binder. Reproduced from Palacín (2009) with permission from the Royal Society of Chemistry.

Although Li-ion batteries (LIBs) dominate the portable electronics battery market, there is a broad diversity of alternative technologies that are successfully used in more niche applications, providing different figures of merit in terms of energy density, costs, lifetime, etc. The perspective of a novel or old battery technology to embracing large-scale applications, such as the grid and renewable solar and wind power, motivates the many current paths of new battery chemistries that can supersede/complement LIBs. One of the plausible solutions is to develop multivalent (Mg, Ca, Al) batteries which, in contrast to LIBs, would be based on the use of metal anodes (Canepa et al., 2017). If successful, this concept would yield leaping breakthroughs in energy density while at the same time being based on cheaper and more abundant elements. Until now, extensive efforts have been dedicated mainly to Mg-batteries. However, electrolyte issues—such as limited electrochemical stability windows (Lipson et al., 2016)—and the lack of operational cathode materials have considerably slowed down the progress in the field (Yoo et al., 2013). In stark contrast, reversible Ca electrodeposition has only recently been unveiled, thereby opening new research avenues (Ponrouch et al., 2016). Ca metal anode-based batteries would enable large gravimetric- and volumetric-specific energies, but this new technology is held back by the limited range of suitable electrolytes and cathodes despite the recently witnessed and significant technical breakthroughs (Gummow et al., 2018; Ponrouch and Palacin, 2018). Before Ca-based batteries can enter the market, electrolyte compositions are required to have electrochemical stability windows over 4 V and enable Ca^2+^ solvation through weak coulombic interactions, improving the overall kinetics and de-solvation at the cathode surface. On the other hand, to overcome sluggish solid-state diffusion, cathode materials should be developed with low migration barriers for calcium ions. The aim of this paper is to quantify the figures of merit attainable with this technology using reliable techno-economic models. Although the Battery Performance and Cost (BatPaC) model (Nelson et al., 2011, 2012) was elegantly and comprehensively applied to Mg batteries (Canepa et al., 2017), no similar reports have tackled Ca batteries. Much simpler than BatPaC, which always considers the full battery pack, the energy-cost model developed by Berg et al. (2015) is employed in this study, considering the performance at the single electrochemical cell level (Figure 1). Hence, we avoid any possible and uncertain differences in electric connections and pouch packaging, and instead the input parameters needed are mainly operating potentials and specific capacities of the active electrode materials.

Here we estimate the energy density of a set of hypothetical full Ca electrochemical cells by modifying the anode configuration, cathode specific capacities, and operating voltage. Furthermore, the results obtained will be compared to the state-of-the-art LIBs (Nitta et al., 2015), Na-ion batteries (SIBs) (Ponrouch et al., 2013; Hwang et al., 2017), and Li/Ca-sulfur (Li-S/Ca-S) battery technologies (Bruce et al., 2011; Hagen et al., 2015), as well as to a hypothetical Ca-ion battery, with graphite as an alternative anode to Ca metal. Finally, some figures regarding cost will be drawn taking LiNi_0.33_Mn_0.33_Co_0.33_O_2_ (lithium nickel manganese cobalt oxide, or NMC)/graphite state-of-the-art LIB technology as a reference (Shaju and Bruce, 2006), by first determining Ca configurations that can compete in terms of energy density and then simulating different possible cathode and Ca metal anode costs. In addition, hypothetical Ca-based systems with an identical operation voltage (i.e., 3.5 V) are considered based on the conditions required to have similar cost. Overall, all these energy and cost estimates serve to survey the future market viability of a Ca metal anode battery (CaB) technology, including selected cathodes, for which reliable data at the laboratory scale cell level are available.

## Results and Discussion

**Figure 2:**
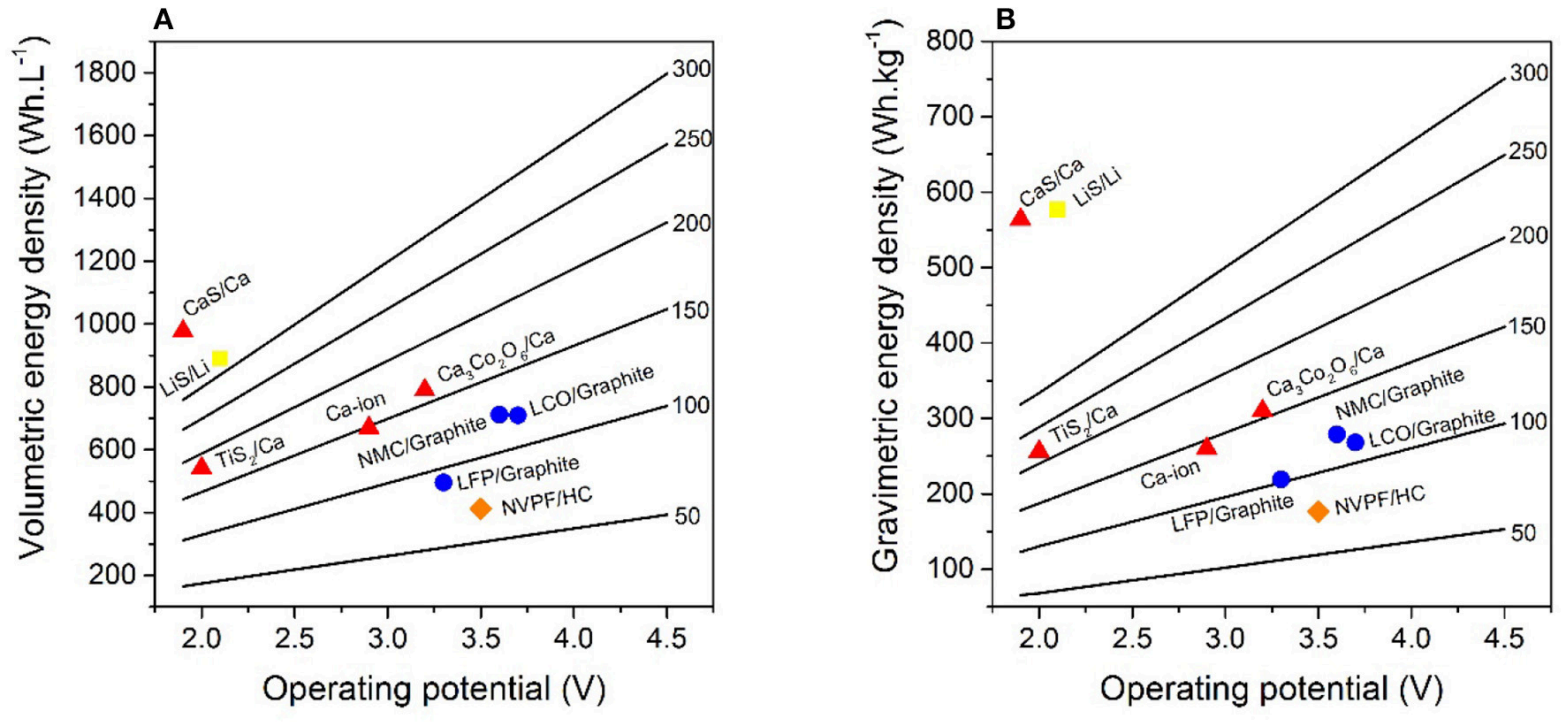
Volumetric **(A)** and gravimetric **(B)** energy densities for LIBs (circle), SIBs (diamond), Li-S (square), and considered Ca (triangles) battery technologies. The straight lines are calculated energy densities of hypothetical CaBs as a function of operation potential and capacities (denoted on the right of each line). All calculations were made using the model developed by Berg et al. (2015).

Table 1 displays active material properties such as average discharge potential and specific capacity used to estimate energy densities, which are subsequently normalized per volume or weight of the studied cell configuration, including current collectors, separator, and electrolyte as well as electrode materials. In addition, the electrode compositions are specified for LIBs, SIBs, CaBs, Li-S, and also Ca-S batteries. LIBs use an aluminum current collector at the cathode, but copper at the anode, as Al alloys with Li at potentials lower than 0.6 V vs. Li^+^/Li. However, for SIBs Al can be used for both current collectors (as Na does not alloy with Al) (Ponrouch et al., 2013, 2016), and this may also be the case for CaBs. The electrode coating thicknesses were adjusted to 100 μm for the cathode to reach a 1:1 charge balancing—c.f. the thickness range for graphite and Ca metal in Table 1—and for electrolyte amount and density the same as for LIBs in Berg's model were used. Two anode configurations have been tested for the hypothetical Ca battery: a 50% Ca excess with Al current collector (denoted conf. 1 in Table 1), mimicking Berg's assumptions for the Li metal anode, and a 100% Ca excess without Al current collector (denoted conf. 2). This excess is to compensate for the potential loss of Ca consumed upon formation of a passivation layer, as is the case for the solid electrolyte interphase (SEI) creation in LIBs. In the second configuration, a larger excess is considered necessary to ensure proper mechanical strength of the anode in the absence of any additional current collector. Calculations were performed for hypothetical CaB cathodes with specific capacities and operating voltages ranging between 50–300 mAh.g^−1^ and 2.0–4.5 V, respectively. Such voltages should be possible by employing electrolytes composed of, for example, Ca(BF_4_)_2_ in a (1:1) mixture of ethylene carbonate (EC) and propylene carbonate (PC) (Ponrouch et al., 2016) or Ca(BH_4_)_2_ in tetrahydrofuran (THF) (Wang et al., 2017), with oxidation stabilities of 4.0 V and 3.0 V, respectively. The difference in energy density between both the two anode configurations is minor, both per volume and weight, <5% even for the most extreme case—i.e., 4.5 V and 300 mAh.g^−1^–which results in 1,797 Wh.L^−1^ vs. 1,719 Wh.L^−1^ and 755 Wh.kg^−1^ vs. 750 Wh.kg^−1^. Therefore, the configuration using a current collector, as is the standard for LIBs, was used. Figure 2 depicts operation potential vs. gravimetric and volumetric energy density for state-of-the-art LIBs as well as SIBs, and also shows the hypothetical CaB, Ca-ion, Li-S, and Ca-S batteries. Specific battery technologies are depicted with symbols, while the hypothetical CaBs are represented by the straight lines with energy densities as a function of the operating voltage and the specific capacity of the cathodes—the capacities are specified on each line. Two specific CaBs have also been considered in more detail: using TiS_2_ and Ca_3_Co_2_O_6_ as cathode materials, both were shown to be electrochemically active at 100°C (Tchitchekova et al., 2018a,b), although full reversibility and cyclability remain to be demonstrated. The hypothetical Ca-ion technology enlists Ca_3_Co_2_O_6_ as the cathode and a graphite anode.

**Table 1:** Active materials and their properties in our cell designs.

	**Average discharge potential** **(V vs. Li/Na/Ca)**	**Capacity (mAh.g^−1^)**	**Density (g.cm^−3^)**	**Volumetric Expansion** **(%)**	**Porosity** **(%)**	**Thickness (μm)**	**Electrode capacity (mAh.cm^−3^)** **incl. Carbon+Binder**
**CATHODES**							
LiCoO_2_ (LCO)	3.8	150	5.05	–	30	104	447
LiNi_0.33_Mn_0.33_Co_0.33_O_2_ (NMC)	3.7	170	4.75	–	30	103	454
LiFePO_4_ (LFP)	3.4	160	3.65	–	30	100	309
Na_3_V_2_(PO_4_)_2_F_3_ (NVPF)	3.8	105	4.00	–	30	100	256
Ca_3_Co_2_O_6_	3.2	160	4.52	–	30	100	435
TiS_2_	2.0 (Ca)	239	3.25	–	30	100	486
Li_2_S	2.1	1000	1.66	80	30	100	850
CaS	1.9	1000	2.59	80	30	100	1152
Hypothetical calcium cathode	2.0-4.5	50-300	4.5	–	30	100	135-810
**ANODES**							
Graphite (Li)	0.1 (Li)	360	2.20	10	36	66-100	466
Graphite (Ca)	0.1 (Ca)	744	2.20	10	36	45	923
Hard carbon (HC)	0.3 (Na)	270	2.00	–	30	73	352
Li metal	0.0	3884	0.53	50	67	61	1383
Calcium metal (conf. 1)	0.0	1338	1.57	50	67	10-58	1400
Calcium metal (conf. 2)	0.0	1338	1.57	100	50	13-77	1050
**OTHER COMPONENTS**							
Aluminum CC			2.7			10	
Copper CC			8.96			40	
Separator+electrolyte			1.02		30 (sep.)	25	
**ELECTRODE COMPOSITIONS**							
Standard	93 wt% Active material / 4 wt% Carbon additive / 3 wt% Binder						
Sulfur	60 wt% Sulfur / 30% Carbon / 10 wt% Binder						

Starting with Ca_3_Co_2_O_6_/Ca, the theoretical energy densities for the resulting CaB would top both state-of-the-art LIB and SIB technologies while being cheaper because of the lower cost of Ca (3 $/ton) (Lime, 2015) compared to Na (10 $/ton) (Sodium, 2015) and Li (15,000 $/ton) ores (Lithium, 2015). Indeed, the Ca_3_Co_2_O_6_/Ca CaB would be ~80 Wh.L^−1^ and 30 Wh.kg^−1^ better than NMC or LiCoO_2_ (LCO)/graphite LIBs, mainly due to the larger energy density of the Ca metal anode compared to graphite (as cathode capacities, densities, and operation potentials are similar). Turning to TiS_2_/Ca, this CaB arrives at 250 Wh.L^−1^ and 55 Wh.kg^−1^ worse than Ca_3_Co_2_O_6_/Ca because of the lower operation potential and higher cathode density, and is also 170 Wh.L^−1^ below both LCO or NMC/graphite LIBs. However, the TiS_2_/Ca CaB is better yet than both LiFePO_4_ (LFP)/graphite LIBs and Na_3_V_2_(PO_4_)_2_F_3_ (NVPF)/HC (Ponrouch et al., 2013) SIBs. In the case of the hypothetical Ca-ion, the composition CaC_6_ was considered for the anode, which has been achieved by chemical reduction of graphite in the presence of calcium (Cahen et al., 2013). The operating voltage is adjusted by the 0.172 V difference in standard reduction potential between the Li^+^/Li and Ca^2+^/Ca couples. As expected, the energy densities of the hypothetical Ca-ion concept are much lower than the Ca metal concept: 120 Wh.L^−1^ and 50 Wh.kg^−1^. This originates from the somewhat lower cell potential, but foremost from the lower energy density related to use of graphite. However, the configuration would still yield higher energy densities than both LFP/graphite and NVPF/HC configurations.

Furthermore, using the values for Li-S batteries, with Li metal anodes calculated by Berg et al. as a starting point, we targeted an analogous Ca-S battery. The energy densities of such a Ca-S battery, together with the Li-S battery, are clearly higher than for any other technology considered here as both the anode and the cathode have very high capacities. However, Ca being denser than Li results in the Ca-S battery having a higher volumetric energy density but lower gravimetric energy density. Hence, there may still be advantages for large-scale applications because of the lower cost of Ca-S as compared to Li-S. There are also many applications where the volumetric energy density is just as precious, or more so, than the gravimetric when a certain threshold on the latter has been achieved.

Overall, CaBs with only moderate operating voltages of 2.1 or 2.5 V and cathode capacities of 250 or 200 mAh.g^−1^ would already yield higher energy densities than the best state-of-the-art LIB. Moreover, in specific cases such as 3.0 V/250 mAh.g^−1^ or 3.5 V/200 mAh.g^−1^, CaBs would have volumetric energy densities above 1,000 Wh.L^−1^, hence higher than any of the sulfur cathode-based technologies. For the gravimetric energy densities, 3.5 V/300 mAh.g^−1^ or 4.0 V/250 mAh.g^−1^ are necessary to supersede Li-S and Ca-S.

**Figure 3:**
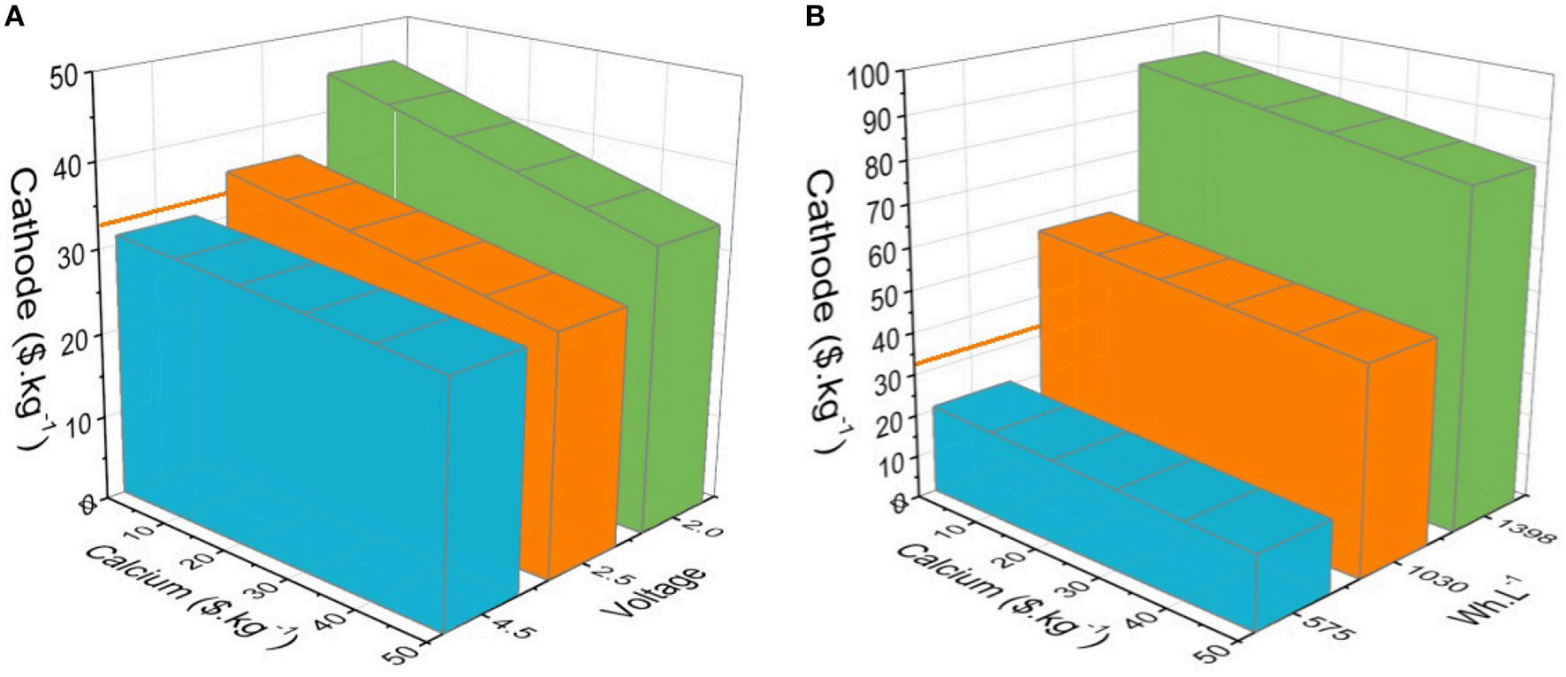
Cost-effectiveness of estimated CaBs with cathode capacities of 100 (blue), 200 (orange), and 300 (green) mAh.g^−1^ vs. a reference NMC/graphite cell (110 $.kWh^−1^) taken as areas representing the costs of both cathode and anode (Ca metal), allowing a total cell cost equal to or lower than 110 $.kWh^−1^ for: **(A)** CaBs with energy densities similar to or higher than NMC/graphite, and **(B)** with an operation potential of 3.5 V, identical to NMC/graphite. The orange line represents the NMC price proposed by Berg et al. (33 $.kg^−1^).

Second in importance, the cost-effectiveness of the hypothetical CaBs is calculated and compared to NMC/graphite. For the latter the cost of both electrodes, current collectors (Cu and Al), separator, and electrolyte, yielding a total of 110 $.kWh^−1^, were taken from Berg et al. Two approaches have been used: (1) cathode capacities fixed at 100, 200, and 300 mAh.g^−1^ and the operation voltage varied to match the calculated energy densities of the NMC/graphite cell (279 Wh.kg^−1^. 711 Wh.L^−1^), resulting in 4.5, 2.5, and 2.0 V, respectively; and (2) the operating voltage fixed to 3.5 V—identical to the NMC/graphite cell—and cathode capacities varied at 100, 200, and 300 mAh.g^−1^ (Figure 3). As the electrode costs vary, the colored areas in Figure 3 represent the anode/cathode pairs with prices allowing CaBs to be either equal or lower in cost than NMC/graphite (110 $.kWh^−1^). The cathode cost is the major factor given the much higher capacity of the Ca metal anode. By fixing the voltage to 3.5 V, the energy densities in general supersede NMC/graphite (711 Wh.L^−1^ and 279 Wh.kg^−1^), only the CaB using a 100 mAh.g^−1^ cathode yielded lower energy densities and therefore limit the cathode cost to a maximum 21 $.kg^−1^ (Figure 3B). On the other hand, the CaB configurations using 200 and 300 mAh.g^−1^ cathodes yield much higher energy densities-−1,030 and 1,398 Wh.L^−1^, respectively—enabling cathode costs of 50 and 80 $.kg^−1^, significantly higher than the cost of NMC (33 $.kg^−1^) (Berg et al., 2015).

## Conclusions

Our simple estimate exercise confirms CaBs to be in principle able to compete with the state-of-the-art LIBs, and this depends to the utmost on Ca metal anodes being viable. In contrast, Ca-ion batteries would fall short in figures of merit for performance and would only be advantageous in terms of cost. If sulfur cathodes were viable, coupling them to Ca metal anodes would provide the best figures of merit for performance, similar to Li-S batteries. Given the relatively high price of Li metal (in addition to imminent production and resource issues), cost and resource advantages for a Ca-S battery are foreseen. Overall, in terms of cost, CaBs are on par with NMC/graphite, even for very high cathode costs (>80 $.kg^−1^).

Despite the technical breakthroughs needed, such as the development of efficient Ca metal anode/electrolyte combinations operating at room temperature and cathode materials, CaBs are clearly a research avenue worth pursuing as, if these breakthroughs are achieved, this future emerging technology would be advantageous from a techno-economic point of view.

## Author Contributions

DM: calculation on performances and data on electrode materials. AP: discussion of results on materials and electrolytes. RA: data on electrolytes and discussion of results. FB: discussion of results on global performance. PJ: design of study and discussion of results. MP: design of study and discussion of results.

### Conflict of Interest Statement

FB is an employee of Toyota Motor Europe company. The remaining authors declare that the research was conducted in the absence of any commercial or financial relationships that could be construed as a potential conflict of interest.
